# Multifractality of Complex Networks Is Also Due to Geometry: A Geometric Sandbox Algorithm

**DOI:** 10.3390/e25091324

**Published:** 2023-09-11

**Authors:** Rafał Rak, Ewa Rak

**Affiliations:** 1 Institute of Physics, College of Natural Sciences, University of Rzeszów, Pigonia 1, 35-310 Rzeszów, Poland; 2 Institute of Mathematics, College of Natural Sciences, University of Rzeszów, Pigonia 1, 35-310 Rzeszów, Poland; erak@ur.edu.pl

**Keywords:** complex networks, fractal networks, models of complex networks, universality

## Abstract

Over the past three decades, describing the reality surrounding us using the language of complex networks has become very useful and therefore popular. One of the most important features, especially of real networks, is their complexity, which often manifests itself in a fractal or even multifractal structure. As a generalization of fractal analysis, the multifractal analysis of complex networks is a useful tool for identifying and quantitatively describing the spatial hierarchy of both theoretical and numerical fractal patterns. Nowadays, there are many methods of multifractal analysis. However, all these methods take into account only the fact of connection between nodes (and eventually the weight of edges) and do not take into account the real positions (coordinates) of nodes in space. However, intuition suggests that the geometry of network nodes’ position should have a significant impact on the true fractal structure. Many networks identified in nature (e.g., air connection networks, energy networks, social networks, mountain ridge networks, networks of neurones in the brain, and street networks) have their own often unique and characteristic geometry, which is not taken into account in the identification process of multifractality in commonly used methods. In this paper, we propose a multifractal network analysis method that takes into account both connections between nodes and the location coordinates of nodes (network geometry). We show the results for different geometrical variants of the same network and reveal that this method, contrary to the commonly used method, is sensitive to changes in network geometry. We also carry out tests for synthetic as well as real-world networks.

## 1. Introduction

The mathematical modeling of networks dates back to the late 1950s, when Erdos and Rényi initiated the field of random graphs [1] and scientists began to develop it. Mathematically, a network is a representation of a real complex system and is defined as a collection of nodes (vertices) and links (edges) between pairs of nodes. Complex networks have naturally become a convenient tool for studying complex systems where many elements (nodes) are observed and connected to each other by a certain interaction (edge). It turns out that one of the greatest challenges in the world of science is the precise and complete description of complex systems, and network research has become an important and indispensable element of this process. Due to their usefulness in studying real-world complex systems, the study of complex networks and multifractal analysis has been developed in many fields such as mathematics, physics, and chemistry [2,3,4]; biological systems [5,6,7,8,9]; economics [10,11]; computer science [12,13,14,15,16,17]; language and sociology [18,19,20,21,22]; and geology [23].

Most real networks show interesting topological features, and their inter-node connections are complex in nature. Often, the study of their characteristics boils down to the analysis of: the assortativeness between vertices, clustering coefficient, degree distribution, reciprocity, centrality, or shortest paths. It seems that a useful and promising tool for identifying the possible complexity of a network structure is fractal analysis, which has been one of the most dynamically developing topics in the last two decades. Moreover, it has been noticed that complex structures can be more effectively characterized in the process of multifractal analysis, where a function of dimensions is replaced by a non-trivial resultant of single fractal dimensions. One of the most important findings in physics was the description of fractal geometry by Mandelbrot [24]. While these beautiful geometric fractal structures apply to structures in physical space, new forms of fractality have been observed in networks, which are the result of complex interactions between nodes. Nowadays, it is assumed that a network’s fractality is the result of its individual or resultant properties and mechanisms such as: self-similarity, the growth phenomenon, the small-world phenomenon, scale-free degree distribution, and self-organization [25,26,27,28,29,30,31,32].

There are two main types of methods for identifying the (multi)fractal properties of complex networks: cluster-growing methods and box-covering methods. Classical box-covering algorithms are focused on solving the problem of how to cover the whole network with the minimum number of boxes, which is related to the family of NP-hard problems (non-deterministic polynomial time) [33]. This family of algorithms includes compact box burning [33], maximum excluded mass burning [34], fixed-size box counting [35,36], and random sequential box covering [37,38]. Cluster-growing methods are an alternative to the traditional box-counting (or -covering) methods. They are based on the method proposed in [39,40]. To put it simply, this method resembles a sandbox and focuses on the scaling of the masses versus the size through sandboxes growing from randomly selected centers. To avoid ambiguity in the results due to the randomness of the method, calculations are repeated for many nodes (seeds) and then averaged. The extension of the sandbox method for the multifractal analysis of complex networks was proposed by Liu et al. [41], which supported the claim that the sandbox algorithm is more accurate than box-counting algorithms for calculating multifractal parameters. This method has also been improved and extended to weighted networks [42,43,44,45].

Characterizing the complexity of a network is very important, e.g., because the functions of a network that represents a given system are the result of its structure. For example, the structure (geometry and relative positions) of an airport connection network affects its efficiency; the structure of the network of synaptic connections in the brain affects its efficiency; and the position of atoms in a material affects the chemical and mechanical properties of the material. Therefore, it should be emphasized that neither the box-covering method nor the sandbox method take into account the network parameters (e.g., the fact that nodes are connected) and their coordinates in space and positions in relation to each other (geometry). In order to overcome the above issues, in this paper we propose a method for the multifractal analysis of complex networks based on both box-counting and sandbox methods.

## 2. Sandbox Algorithms

### 2.1. The Standard Sandbox Algorithm

**Figure 1:**
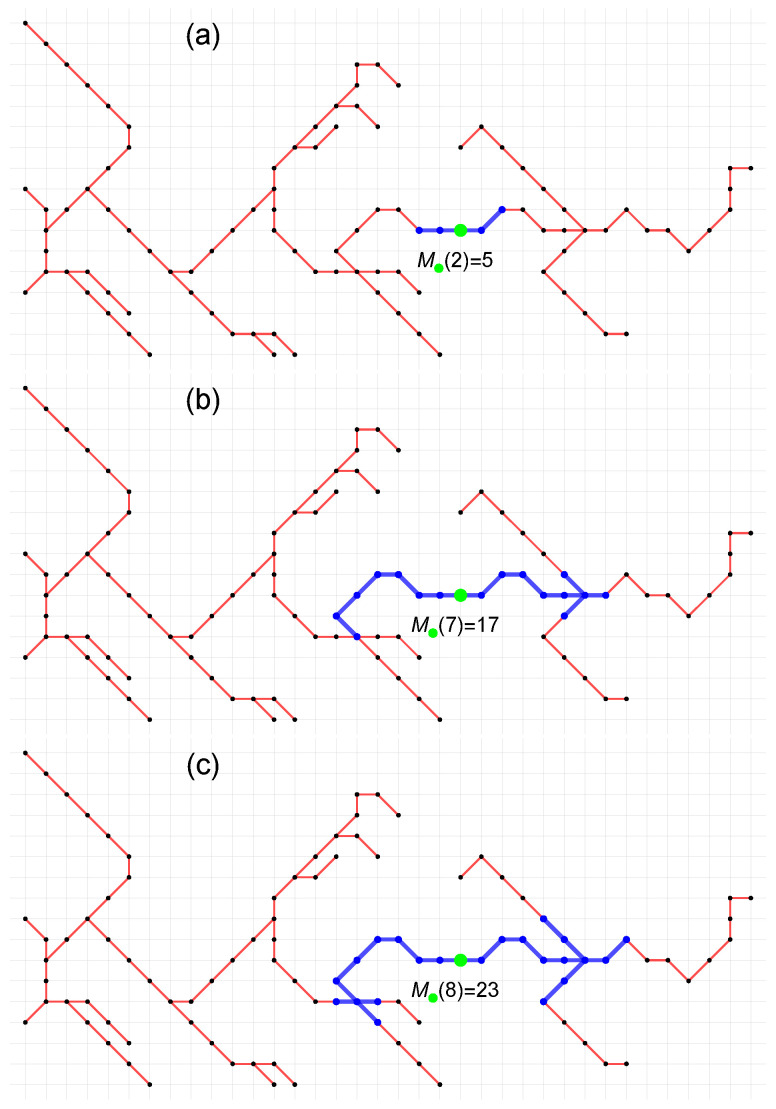
The standard sandbox algorithm. The number of nodes M(r) covered by the sandbox with the same center node for three different radius values r={2,7,8}.

The idea and main steps of the existing sandbox algorithm (SB) for the multifractal analysis of network *Q* can be described as follows:

(1) The set of the radius r(1≤r≤d) of the sandbox is determined, where *d* denotes the diameter of the network *Q*.
(2) A node set C(r) is randomly chosen as the centers of the sandboxes.
(3) The number of nodes $M_{i}\left(r\right)$ covered by the sandbox with the center node i∈C(r) and radius *r* is counted. This process for one of the center nodes *i* and r={2,7,8} is illustrated in Figure 1a–c. It should be emphasized that *r* is not a geometric radius but a measure of the propagation over the closest neighboring nodes of the *Q* network.
(4) For each scaling parameter q≠1, the average $\langle {\left[M_{i}\left(r\right)\right]}^{q-1}\rangle$ over all sandboxes of radius *r* is calculated.
(5) For a fixed set of radii selected from 1≤r≤d, steps (2)–(4) are repeated.
(6) If linear dependence between $\ln(\langle {\left[M_{i}\left(r\right)\right]}^{q-1}\rangle )/\left(q-1\right)$ and ln(r/d) is observed, then one can refer to the (multi)fractal nature of the investigated network *Q* and calculate the reliable generalized fractal dimension $D_{q}$ and the singularity spectrum f(α) as follows:
  (1) $$D_{q}=\lim_{r\to 0}\frac{\ln\langle {\left[M_{i}\left(r\right)\right]}^{q-1}\rangle }{\ln(r/d)}\frac{1}{q-1},\;\;q\in ℜ,\;\;q\neq 1,$$
  (2) $$\tau_{q}=\left(q-1\right)D_{q};\;\;\;\;\alpha =\frac{d}{dq}\tau_{q}\;\;\mathrm{and}\;\;f\left(\alpha \right)=q\alpha -\tau_{q},$$
  where α is called the singularity (Hölder) exponent. The wealth of multifractality present in the analyzed *Q* network can be defined as both the spread of the generalized fractal dimension $D_{q}$: $\Delta D_{q}=D_{q_{min}}-D_{q_{max}}$ and the width of the multrifractal spectrum f(α): $\Delta \alpha =\alpha_{max}\left(q_{min}\right)-\alpha_{min}\left(q_{max}\right),$ where $q_{min}$ and $q_{max}$ are, respectively, the minimal and the maximal values of the deformation parameter *q*.

### 2.2. The Geometric Sandbox Algorithm

**Figure 2:**
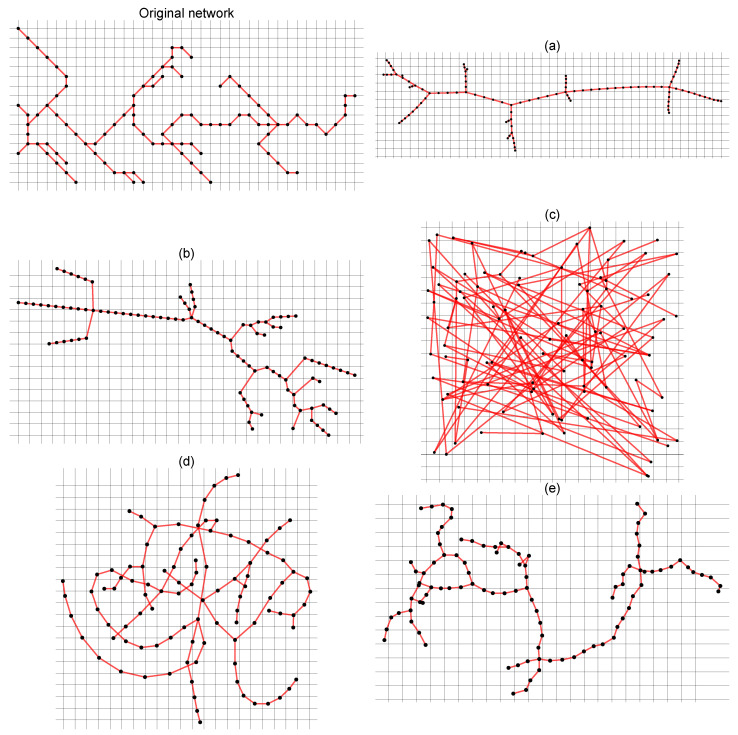
The same network in different geometrical representations (**a**–**e**) where nodes have the same structure of connections and degree but are distributed differently in relation to each other in the plane. In each case, only the geometric coordinates of the location of each node change, and thus the length of the edge.

**Figure 3:**
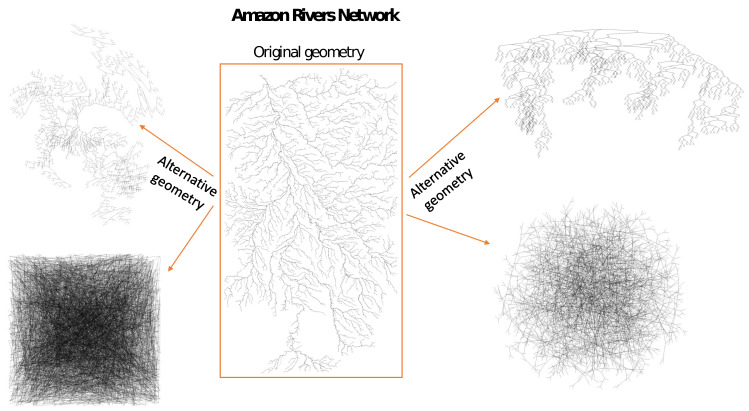
Alternative geometries of the real tributary network of the Amazon River.

**Figure 4:**
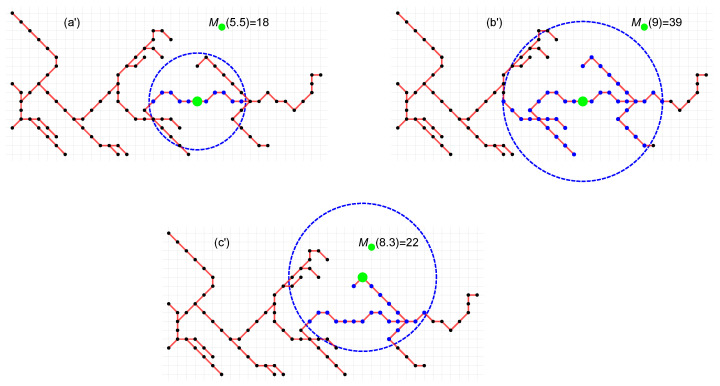
An example of the counting of network nodes in the GSB method for 3 radii *R* of a circle. Examples (**a′**) and (**b′**) use two different circle radii R={5.5,9} for the same central node. Example (**c′**) shows the counting of nodes for a different central node and radius R=8.3.

Let us consider the same network in different geometrical representations where the nodes have the same structure of connections but are distributed differently in relation to each other in the plane (Figure 2 and Figure 3). Changing the position of the nodes changes the length of the edges but does not change the nodes’ degree. Without taking into account the weights between nodes, from the network point of view, these networks are identical—for each of them, the result of the standard SB algorithm is identical. However, our intuition tells us that these networks may (and even should) have different characteristics of complexity. Of course, there is a sandbox version of the algorithm that takes into account the weights of connections between nodes where the weight can be, e.g., the distance between two neighboring nodes. However, this is just an interaction between neighbors, and more global peer-to-peer interactions are not considered. In order to take into account both the geometric position of the nodes and their network properties in the multifractal analysis, we propose a modified SB algorithm—the geometric sandbox algorithm (GSB). The next steps of the GSB algorithm are as follows:

(1) The set of the radius of circles R(0<R≤P) of the sandbox is determined, where *P* denotes the geometric diameter (the distance between the most distant nodes) of the network *Q*. In practice, however, we choose the smaller R(0<R≤P/5). Unlike the standard SB, here the radius *R* is not, in general, an integer number (expressed as multiples of the edge), but is a geometric distance (a real number).
(2) A node set C(R) is randomly chosen as the centers of the sandboxes.
(3) The number of nodes $M_{i}\left(R\right)$ covered by the sandbox with center node i∈C(R) and radius *R* of the circle is counted. The number $M_{i}\left(R\right)$ does not always include all nodes within the circle. As Figure 4 shows, the number $M_{i}\left(R\right)$ includes only those nodes that form a connected graph with node *i* (nodes marked in black, although they are in a circle, are not included in $M_{i}\left(R\right)$). Counting only nodes that form a graph connected to node *i* ensures that we do not include nodes in a circle of radius *R* that do not directly interact with the central node *i* (we do not count nodes from which we cannot reach node *i* along the edges). This approach to counting nodes is a convolution of the classic SB and the box-covering methods: we count nodes from a given box (circles here, though there may also be another flat figure), but only those that have a network connection with the center node. This process for R={5.5,9,8.3} is illustrated in Figure 4. The nodes included in the M(R) number are marked in blue.
(4) Steps (4)–(6) are the same as for the standard SB algorithm.

## 3. Numerical Tests

**Figure 5:**
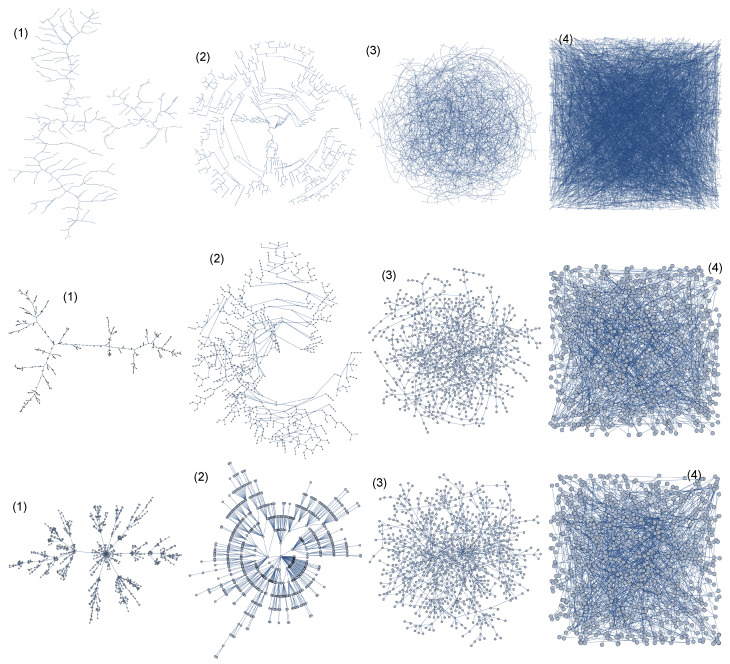
Four variants of geometry ((1)–(4)) for three types of networks: top—a network of Ligurian mountain ridges; middle—a tree network; bottom—a scale-free network. Changing the geometry consisted in changing the position of the nodes on the plane but did not involve changing the nodes’ degree. In order for better visualization, networks with a small number of nodes are shown.

In order to test the possible influence of the network geometry, i.e., the geometric position of the nodes in the network, we considered three networks: the real network of the ridges of the Ligurian mountains, a tree-type network, and a scale-free network. For each type of network, multifractal analysis was performed for four alternative geometries of the same network. Changes in the geometry of each network were made in such a way that the location of the nodes was changed; thus, both the distances between the nearest neighbors and the relative distances between all other nodes were changed (see Figure 2 and Figure 3, where alternative geometries of the Amazon River network are shown). It should be emphasised that changes in geometry do not change the degree of nodes, so the classic SB method, regardless of the geometry (appearance) of the network, gives the same result. Of course, there is a weighted variant of the classic SB method that can take into account the distances between nodes [42], but unlike the GSB method proposed here, the weighted variant of the SB method cannot take into account the relative positions of all nodes. Geometric changes to the network (alternative geometries) were made in the Mathematica software system (the ’GraphM’ package was used for this; ’IGraphM’ is a Mathematica package for use in complex networks and graph theory research. The creator of the interface was Szabolcs Horvát [46]). An example is shown in Figure 5, presenting four different geometric variants of the Ligurian mountain ridges, tree-type network, and scale-free network, respectively: 

(1) IGLayout FruchtermanReingold (the vertices were deployed on a plane according to the Fruchterman–Reingold algorithm) [47];

(2) IGLayoutReingoldTilfordCircular (the vertices were deployed on a plane according to the Reingold–Tilford algorithm) [48];

(3) IGLayoutGraphOpt (algorithm by Michael Schmuhl) [49];

(4) IGLayoutRandom (the vertices were deployed uniformly and randomly on a plane) [49].

To improve the clarity of the figure, the number of network nodes was significantly reduced, which in the case of the mountain ridges meant that only part of the mountain range is shown.

**Figure 6:**
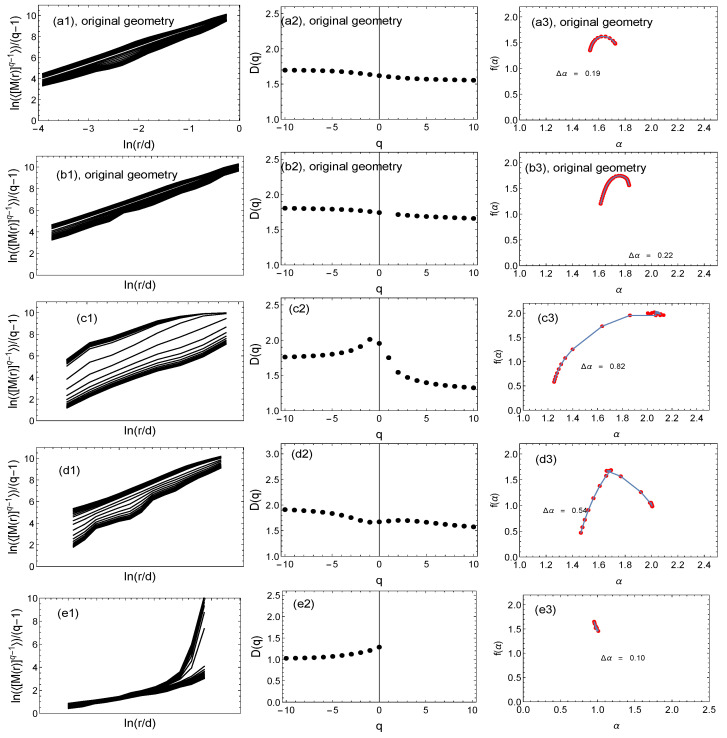
The multifractal analysis using the classic sandbox algorithm (**a1**–**a3**) and its modification, i.e., the geometric sandbox method (other graphs) of the network of Ligurian mountain ridges. The first two rows are the results for the original real network; rows (**c**–**e**) are the results for alternative geometries of the original network using the GSB algorithm. Columns (1), (2), and (3) show fluctuation functions, the fractal dimension, and the multifractal spectrum, respectively.

**Figure 7:**
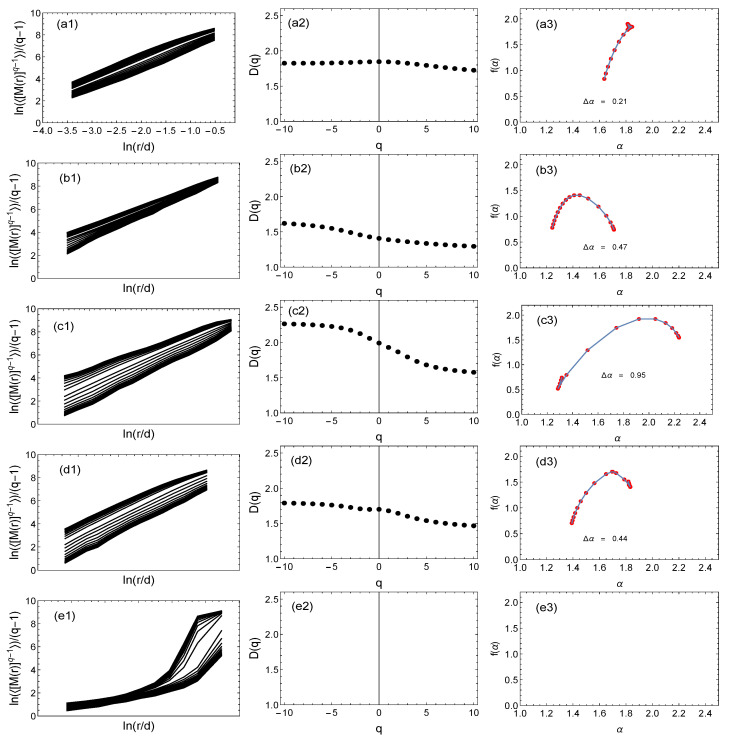
The multifractal analysis using the classic sandbox algorithm (**a1**–**a3**) and its modification, i.e., the geometric sandbox method (other graphs) of a synthetic tree-type network. The rows show the results for alternative geometries of the network. Columns (1), (2), and (3) show the fluctuation functions, fractal dimension, and multifractal spectrum, respectively.

**Figure 8:**
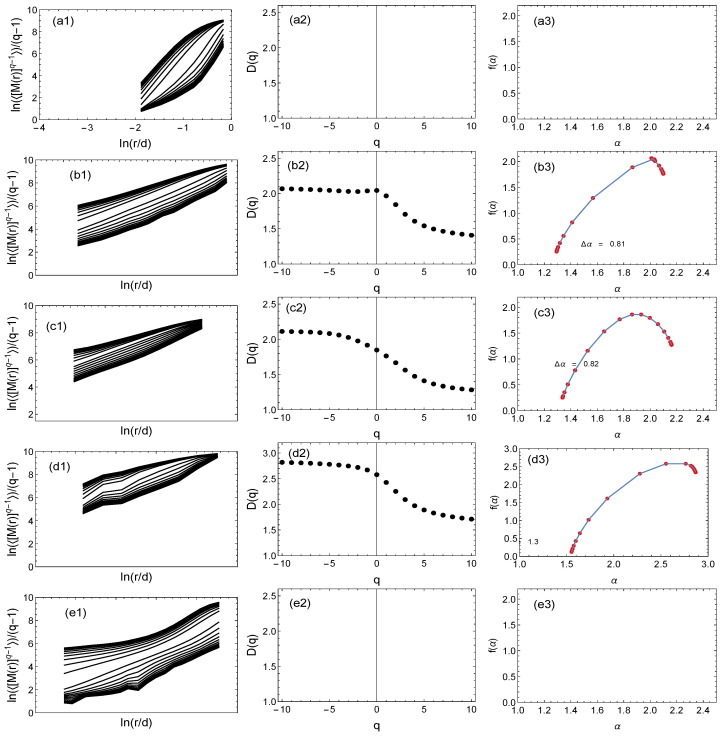
Multifractal analysis using the classic sandbox algorithm (**a1**–**a3**) and its modification, i.e., the geometric sandbox method (other graphs) of a synthetic scale-free network. The rows show the results for alternative geometries of the network. Columns (1), (2), and (3) show the fluctuation functions, fractal dimension, and multifractal spectrum, respectively.

Figure 6, Figure 7 and Figure 8 show the results of the multifractal analysis using the classic SB method and its modification proposed here, i.e., the GSB method. For the tree network (Figure 7) and the scale-free network (Figure 8), the network size was the same at 40,000 nodes. This number was chosen because the number of nodes for the real mountain ridge network was 40,396 nodes (Figure 6). The results for the generated (synthetic) tree and scale-free networks were averaged over 10 independent realizations.

The following notations are used: (a) represents the results for the SB method; (b, c, d, e) are the results for the GSB method. In the case of (a) and (b), a network with the same geometry was analyzed. For an easier comparison of the results, the graphs are presented in the same numerical range. For each network, the fluctuation function $(<{\left[M\left(R\right)\right]}^{\left(q-1\right)}>/\left(q-1\right))\equiv F$ was determined first (graphs with index (1)). Then, if the fluctuation function *F* on the log–log scale was a straight line, we determined the fractal dimension $D_{q}$ and the singularity spectrum f(α) (graphs with index (2) and (3), respectively). In the case of both the real network (Figure 6) and the other two synthetic networks (Figure 7 and Figure 8), it can be seen that changing the network geometry changed the multifractal characteristics. In four cases, the fractal nature of the network was not found because the *F* fluctuation function did not scale in the full range of *q* values (Figure 6e) or there was no scaling in the entire *q* range and *R* radius (Figure 7e and Figure 8a,e). Therefore, in these cases, it was not reasonable to determine subsequent multifractal characteristics.

The SB algorithm for the ridge network of the Ligurian mountains indicated the multifractal nature of the network (Figure 6(a1–a3)), where the width of the multifractal spectrum Δα=0.19.

Figure 6(b1–b3) shows the results of the GSB method for the original network geometry, which was geometrically similar to Figure 5 (1). It can be seen that taking the geometry into account did not significantly affect the multifractal characteristics, i.e., Δα=0.22. We only observed a slight shift of the whole spectrum to the right. The next three rows of graphs in Figure 6 show the results of the GSB algorithm for three different geometries—similarly to the first row in Figure 5 ((2)–(4)). It can be seen that the change in the real network geometry of the mountain ridge entailed a change in the multifractal characteristics, and the widest spectrum was observed for cases (c) and (d). In the case of (e), only the fractal dimension $D_{q}$ and the multifractal spectrum f(α) for q<0 are shown because scaling in the full range of scales was observed only for these values. Furthermore, for this type of geometry (analogous to Figure 5 (4)), where the nodes were randomly distributed, the shift of the whole spectrum towards α=1 was visible, suggesting the depletion of the complexity of the network, and elements of the monofractal nature of the network could only be seen for q<0.

Figure 7a–e shows the results for a synthetic tree network with the geometric representations shown in Figure 5 (middle row 1–4). The results of representation (1) are shown in a1, a2, and a3 (SB) and b1, b2, and b3 (GSB). It should be noted that example (1) was qualitatively similar to the real ridge geometry of the Ligurian mountains; therefore, the results were similar to the results for the original mountain geometry. For geometries (2) and (3), we could also see the influence of the geometry on the fractal nature of the lattice (Figure 7c,d). For geometry (2), the spectrum was the widest and most shifted to the right. In the case of (e), for geometry (4), where the nodes were arranged randomly, similarly to the real network, we observed a lack of scaling of the fluctuation function in the interval *r* long enough to confirm the fractal nature of this network. Insignificant scaling elements could be seen only in a short range of small lengths *r*. Therefore, no attempt was made to estimate $D_{q}$ and f(α), suggesting that this type of network with such a geometry is not fractal.

Figure 8a–e show the results for a synthetic Barabasi–Albert-type scale-free network. In this type of network, the degree distribution has a low-power nature, which may suggest the fractality of this network. However, the classic SB method suggested quite the opposite (Figure 8a)—the fluctuation function did not scale, so the fractal dimension $D_{q}$ and the multifractal spectrum f(α) did not exist. However, when we applied the GSB method taking into account the geometry of the network, fractality appeared and, as in the previous examples, was dependent on the type of geometry (Figure 8b–d). In cases (b–d), the *F* fluctuation function was exponential in the whole range *r*, and the multifractal spectra were relatively wide (Δα took values of 0.81, 0.82, and 1.3). The values of Δα that were significantly higher than zero testified to the complex nature of these networks. For geometry (e), where the nodes were distributed randomly (in the geometrical sense), similarly to the previously discussed types of network, the fluctuation function was not low-power; therefore, it was not justified to calculate other fractal characteristics.

## 4. Conclusions

This article presented a different approach to the study of the multifractality of complex networks. For this purpose, we proposed a modification of the sandbox method. The geometric sandbox takes into account both connections between nodes and the location coordinates of nodes (network geometry). This approach combines both a typical sandbox network approach (only considering nodes connected to each other) and a box-counting approach (only including nodes lying within a circle of radius R). We tested our method for different geometrical variants of the same synthetic and real networks. It has been shown that networks with a geometry that is pleasing to the human eye have wider multifractal spectra, in contrast to those where the nodes are randomly distributed (these types of network are not fractal). The test results confirmed that the complexity of the network (its multifractal characteristics) is sensitive to changes in its geometry (the coordinates of nodes).

Therefore, it seems that the method proposed here could be a useful tool for identifying and studying the degeneration of network multifractal structures in the case of not only 2D but also 3D networks, where geometry often plays an important role—for example, the study of changes in DNA structures or brain neural networks.
